# Aligning training, regulation, and payment policy to advance the behavioral health workforce

**DOI:** 10.1093/haschl/qxae148

**Published:** 2024-11-13

**Authors:** Brianna Lombardi, Lisa de Saxe Zerden, Erin Fraher

**Affiliations:** Behavioral Health Workforce Research Center, Cecil G. Sheps Center for Health Services Research, University of North Carolina at Chapel Hill, Chapel Hill, NC 27599, United States; Department of Family Medicine, School of Medicine, University of North Carolina at Chapel Hill, Chapel Hill, NC 27599, United States; School of Social Work, University of North Carolina at Chapel Hill, Chapel Hill, NC 27599, United States; Behavioral Health Workforce Research Center, Cecil G. Sheps Center for Health Services Research, University of North Carolina at Chapel Hill, Chapel Hill, NC 27599, United States; School of Social Work, University of North Carolina at Chapel Hill, Chapel Hill, NC 27599, United States; Behavioral Health Workforce Research Center, Cecil G. Sheps Center for Health Services Research, University of North Carolina at Chapel Hill, Chapel Hill, NC 27599, United States; Department of Family Medicine, School of Medicine, University of North Carolina at Chapel Hill, Chapel Hill, NC 27599, United States

**Keywords:** health workforce, behavioral health workforce, behavioral health, health policy

## Abstract

The United States is facing an unprecedented behavioral health crisis, exacerbated by workforce shortages that limit access to treatment. In response, states are attempting to increase access to behavioral health services by developing new professions and roles and expanding the functions of the existing behavioral health workforce. Yet, training, regulation, and payment policies are often not aligned to effectively deploy the workforce to serve in new or expanded roles to meet behavioral health needs. We envision training, regulation, and payment as a three-legged stool that supports the health care workforce. In this commentary, we discuss why each leg of the stool is essential, offer examples of how misalignment occurs in the behavioral health workforce, and provide an example of how states can align these three factors to meet community behavioral health needs.

The United States is facing an unprecedented behavioral health crisis, exacerbated by workforce shortages that limit access to treatment.^1^ Estimates over the last 25 years have consistently reported that only half of those needing mental health care receive it and less than 20% of those who need addiction treatment gain access to services.^2^ The stress and impact of the COVID-19 pandemic brought an increasing need for behavioral health services that has far outpaced the supply of the behavioral health workforce.^3,4^ More than half of the Untited States population lives in a mental health professional shortage area and fewer than 30% of rural counties have access to a psychiatrist.^5^ In response to these persistent and intensifying concerns, states are focused on addressing the behavioral health workforce crisis. Two ways that states are attempting to increase access to behavioral health services are through (1) expanding the functions and roles of the existing behavioral health workforce and (2) developing new types of behavioral health care workers. Yet, efforts to increase workforce capacity through existing and new behavioral health pathways require simultaneous alignment of training, regulation, and payment policy; without all three components in place, the workforce cannot be effectively deployed.

In this commentary, we use the three-legged stool as a conceptual framework to illustrate how training, regulation, and payment are intertwined. As a stool needs all three legs for stability and to function, a workforce cannot be fully deployed if training, regulation, and payment are not concurrently aligned (see Figure 1). We draw on examples from the peer support workforce, nurse practitioners and physician assistants, licensed marriage and family therapists (LMFTs) and licensed mental health counselors (LMHCs), and prescribing psychologists to demonstrate how each component of the three-legged stool impacts the behavioral health workforce. Given that many of the policy levers related to behavioral health training, regulation, and payment of the behavioral health workforce are within states’ purview, these examples can provide state policymakers with a framework to consider when deciding how best to invest in the existing and new behavioral health workforce to meet increasing behavioral health needs.

**Figure 1.**
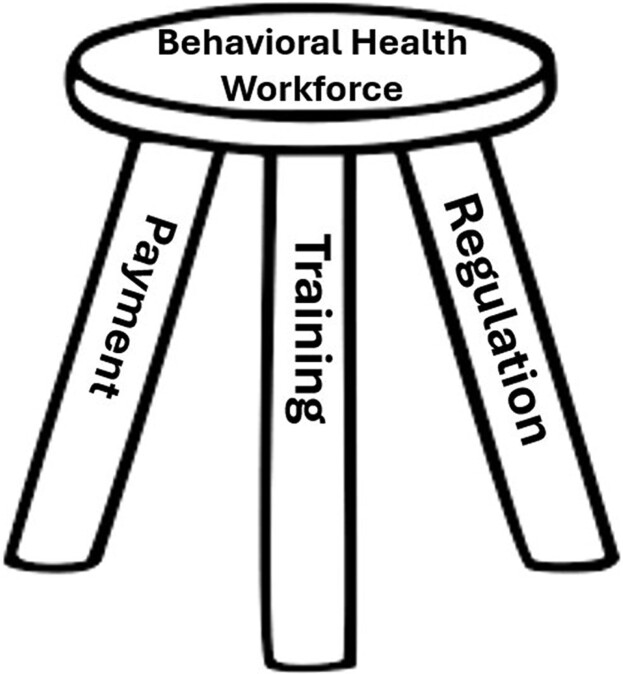
Three-legged stool needed to advance the behavioral health workforce.

## Components of the Three-legged stool

### Training

Behavioral health workforce training requires a curriculum that meets accreditation standards and provides students with core competencies and clinical training opportunities. Behavioral health education must be continually updated to reflect new practice realities and address evolving population needs. Educational bodies must be available to train the workforce to serve in new roles and functions, both through training new workers to enter practice and by providing continuing education to the existing workforce to “retool” their skills to take on new functions.^6^

### Regulation

Health workforce regulation, including state scope of practice (SOP) laws, licensure, and certification, determines the functions and tasks that a health worker can perform. Regulation is important to ensure public safety through disciplining workers who are not meeting practice standards and plays an important role in requiring documentation of competence to enter practice.^7^ To develop a new type of worker or expand the role of an existing workforce, SOP legislation changes may need to occur, and the regulatory board may need to develop and implement new practice standards.

### Payment

The third leg of the stool needed is payment—ensuring that there are reimbursement mechanisms available to pay new and existing behavioral health workers for service delivery. State Medicaid plans and private payors need to incorporate procedure codes that pay for new behavioral health care delivery and service models. This may mean including new behavioral health occupations as eligible billing personnel and allowing for existing behavioral health providers to be reimbursed for a broader set of behavioral services procedure codes.

## Alignment of the training, regulation, and payment in the existing workforce

The demand for behavioral health services has outpaced the supply of workers, and one important way to close this gap is to consider how changes to regulation, payment, or training of the existing workforce would allow behavioral health workers to be more effectively deployed to meet demand.^6^ While most behavioral health professions have national uniformity in training, regulation, and payment (ie, clinical psychologists, clinical social workers, psychiatrists), some behavioral health occupations, largely the ones classified as non-licensed or “paraprofessionals,” have significant variation across states.^8-10^ This variation sometimes leads to a patchwork of policies across all components of the three-legged stool.

### The peer support workforce: an example of alignment

Although peer-delivered services have been used in the behavioral health field since the 1970s and there is significant evidence to support peer support roles in the treatment of behavioral health conditions,^11,12^ alignment of training, regulation, and payment structures across states has only begun to take shape over the last 10 years. Now, most states have developed peer support specialist training competencies required for practice, credentialing systems to certify peer support specialists, and payment mechanisms to reimburse their contributions to behavioral health care. Recognizing the important role of peer support specialists across the United States, Medicare finalized rules in 2024 that created a new mechanism to pay for peer services to address unmet social needs and help patients with high-risk conditions identify and connect with appropriate clinical and support resources.^13^

While training, regulatory, and payment changes have occurred, allowing the peer support workforce to be more effectively deployed to meet demand, there continue to be areas of misalignment within and across the three legs for this workforce. For example, although the Substance Abuse and Mental Health Services Administration (SAMHSA) has set forth national training competencies, training curricula and required competencies for peers to practice vary across states. This variation impacts the portability of peer credentials across state lines.^14^ Even when training and certification are aligned, payment for peer services may be missing. Vermont and Wisconsin have established state-regulated training and certification processes for peer support specialists but do not provide Medicaid reimbursement for peer support services.^15^ Further, of the 48 states that offer Medicaid reimbursement, the types of services reimbursed vary.^15^

### Nurse practitioners: regulation

Even when training and payment are aligned, state regulation may prevent a workforce from performing a role or function that could meet critical behavioral health needs. For example, while nurse practitioner (NP) training is standardized and credentialed through national organizations and NPs can bill for their services, state variation in SOP regulations can impact access to a broad range of behavioral health services, including medications for opioid use disorder (MOUD). Previous studies have demonstrated that fewer MOUD prescriptions are written in counties with restrictive NP practice.^16^ Twenty-six states require NPs to have a collaborative practice with a physician, a requirement that may limit opportunities for NPs to treat patients with a broad range of behavioral health conditions.^17^ This limitation is particularly problematic in rural communities, given that psychiatric mental health NPs are more likely to prescribe in rural communities in states with full practice authority.^18^ Yet, changing regulation and expanding a health workers’ SOP can be stymied by professional organizations and lobbying groups that may be inclined to advocate for their own self-interest^19,20^ over modernizing SOP regulations to prioritize meeting needs.

### LMFTs/LMCHCs: payment

Often, payment is the single most important factor restricting the behavioral health workforce from being effectively deployed. For example, the Centers for Medicare and Medicaid Services (CMS) did not allow LMFTs and LMHCs (i.e., licensed professional counselors) to bill Medicare for psychotherapy until 2024 despite rapid growth of training programs for LMFTs and LMHCs and licensure of these occupations. This meant that, until very recently, LMFTs and LMHCs were not able to independently bill Medicare for services, and thus were an untapped workforce with the potential to meet behavioral health workforce needs, particularly in areas with fewer behavioral health clinicians. A recent analysis found that counselors were more likely than psychologists and social workers to be practicing in underserved areas, despite these occupations not yet being able to bill Medicare.^21^ Current work is underway to examine how allowing LMFTs and LMHCs to bill for service is expanding access to behavioral health services, given the policy change focused on payment.

### Prescribing psychologists: expanded functions

Alignment of each leg of the stool—training, regulation, and payment—takes time, particularly for existing occupations that are expanding roles or responsibilities that overlap with other health professions. An example of an existing behavioral health occupation that has slowly expanded training and changed SOP regulation are prescribing psychologists—doctoral trained licensed clinical psychologists—who prescribe psychiatric medication.^22^ Although prescribing psychologists were first granted prescriptive authority in the early 2000s in New Mexico and Louisiana, it took time to establish the training bodies and curriculum required to train psychologists seeking this additional certification. Expanding the role of psychologists to include prescribing privileges was intended to enhance access to care for behavioral health conditions that require pharmacological interventions.^22^ Currently, seven states allow psychologists to prescribe medications after completing additional training^23^ and six training programs are available, with other training programs in development.^24^ Still, few prescribing psychologists practice within this expanded SOP model and more research is needed to evaluate whether these expanded roles increase access to behavioral health care and needed pharmacological treatment.

## Alignment of the training, regulation, and payment for new occupations

When a state identifies gaps in access to behavioral health care services, new training, regulation, and payment models can be developed and implemented concurrently to allow for new types of behavioral health workers to be deployed. An example of this approach recently occurred in Washington State where they aligned training, regulation, and payment to create a new bachelor's-level role, Behavioral Health Support Specialists, to work in the behavioral health field.^25^ Before this new title, individuals completing their undergraduate degrees had no clear career path to enter the behavioral health workforce. In response, universities in the state of Washington joined together to develop a shared curriculum and competencies for a bachelor's-level behavioral health support specialist.^26^ Next, the state passed legislation to create a new credential for this role and required the health care authority to include behavioral health support specialists in state Medicaid payment plans. This legislation was signed by the governor in Spring 2023 and implementation will occur in 2025 (SSB 5189).^27^

Washington's approach provides a framework for other states to consider when developing new roles and pathways into the behavioral health workforce. Future workforce research needs to assess whether these new pathways increase entry into behavioral health professions and ultimately improve access to behavioral health care in the state. Evidence from the peer support workforce literature suggests that removing barriers to entry, particularly related to formal higher education^28^ and inadequate pay,^14,29^ may increase career laddering in the behavioral health workforce. As states and the nation work to increase behavioral health workforce supply, incentivizing entry into the field from multiple pathways is needed.

## Conclusion

As the behavioral health crisis continues and intensifies, workforce solutions are needed to increase access to behavioral health services. Yet, because the behavioral health workforce is diverse, with many different occupations and skill sets serving different populations, aligning training, regulation, and payment to accommodate new roles and functions is challenging but critical. States should look within their existing behavioral health workforce and examine areas where training, payment, or regulation may be restricting a workforce from meeting the needs of different populations. At the same time, states can look to see if gaps in service provision could be addressed through a new training program, regulatory approach, or payment model. Organizations such as the National Academy for State Health Policy, National Council of State and Legislatures, and National Governors Association could play an important role in collecting and disseminating innovative approaches to training, regulating, and paying the behavioral health workforce across states. Given innovations and changes underway, states can learn from each other to find the appropriate levers needed to strengthen the behavioral health workforce.

## Supplementary Material

qxae148_Supplementary_Data

## Data Availability

Not applicable.
